# Endocrine therapy initiation and overall survival outcomes with omission of radiation therapy in older Medicare patients with early‐stage hormone‐receptor‐positive breast cancer

**DOI:** 10.1002/cam4.5488

**Published:** 2022-11-25

**Authors:** Chan Shen, Ning Li, Shouhao Zhou, Kelly Stahl, Daleela Dodge, Hui Zhao

**Affiliations:** ^1^ Department of Surgery The Pennsylvania State University, College of Medicine Hershey Pennsylvania USA; ^2^ Department of Public Health Sciences The Pennsylvania State University, College of Medicine Hershey Pennsylvania USA; ^3^ Penn State Cancer Institute Hershey Pennsylvania USA; ^4^ Department of Economics and Finance Salisbury University Salisbury Maryland USA; ^5^ Department of Health Services Research University of Texas, MD Anderson Cancer Center Houston Texas USA

**Keywords:** early‐stage hormone‐receptor‐positive breast cancer, endocrine therapy initiation, older breast cancer patients, radiation therapy, SEER‐Medicare database

## Abstract

**Background:**

Guidelines allow for the omission of radiotherapy in older women with early‐stage, hormone‐receptor‐positive breast cancer, given that the patients receive adequate endocrine therapy (ET). However, the initiation of ET and survival outcomes after forgoing radiation therapy among these patients have not been well‐studied.

**Methods:**

We identified patients aged 70 to 90 years old newly diagnosed in 2010–2015 with early‐stage, hormone receptor positive, and human epidermal growth factor receptor 2 (HER2) negative (HR+/Her2−) breast cancer who received lumpectomy and omitted radiation therapy using the SEER‐Medicare database. We examined the initiation of ET and the utilization patterns of ET using a multivariable logistic regression. We further examined the overall survival outcomes using Kaplan–Meier estimation and Cox proportional hazard model with inverse probability weighting.

**Results:**

Of the 2618 patients, 808 (30.9%) received no ET. The multivariable logistic regression showed that more recent years had better ET initiation (2013–2015 vs. 2010–2012: OR = 1.39, 95% CI:[1.16, 1.66]), while older patients (81–90 vs. 70–80: OR = 0.45, 95% CI:[0.38, 0.54]) were less likely to receive ET. Both the Kaplan–Meier estimation (log‐rank p‐value<0.0001) and the Cox proportional hazard model with inverse probability weighting (HR = 0.76, 95% CI:[0.58, 0.99]) showed that receiving ET was associated with better overall survival.

**Conclusion:**

This population‐based study suggests that a sizable proportion of patients who omitted radiation did not receive endocrine therapy and receiving endocrine therapy was beneficial among these patients. Although ET initiation has improved in more recent years, certain patient groups were still especially susceptible to no endocrine therapy.

## Graphical abstract

The overall survival of patients receiving ET was significantly better than patients not receiving it (p‐value<0.0001) based on log‐rank test. We found that patients who received endocrine therapy had significantly better survival than patients who did not (HR=0.76, 95%CI=[0.58,0.99], p‐value=0.04).

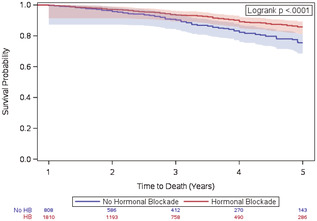

## 1 INTRODUCTION

Several randomized trials have shown that older patients with early‐stage hormone‐receptor‐positive breast cancer are candidates for omission of radiation therapy after receiving breast‐conserving surgery.^1^, ^2^, ^3^ The National Comprehensive Cancer Network guidelines suggest that older patients could consider omitting radiation therapy and only receive adjuvant endocrine therapy post lumpectomy.^4^ Several observational studies have shown the shift in adjuvant treatment pattern among this patient group from radiation therapy or radiation therapy plus ET to ET alone.^5^, ^6^, ^7^, ^8^ Some of these studies are limited by being institutional studies that included a small group of patients receiving treatment at large medical facilities,^9^ and the majority of the results are based on data prior to 2012. More importantly, much less is known about the uptake of endocrine therapy among patients who omitted radiation therapy. Further, few studies have examined the impact on survival outcomes when patients forego ET as radiation therapy was not delivered.

In this study, we aim to fill these three important knowledge gaps and examine the utilization pattern of ET among older breast cancer patients with the American Joint Committee on Cancer (AJCC) Stage I disease who omitted radiation therapy using a large national database. Further, we examine the impact of ET on survival outcomes for these older patients. We hypothesize that a sizable group of patients who omitted radiation also omitted ET, and such double omissions are negatively associated with survival outcomes.

## 2 METHOD

### 2.1 Data source

The data source for the study was the Surveillance, Epidemiology, and End Results (SEER) registry data linked with Medicare claims from the National Cancer Institute (NCI). The SEER cancer registry data are widely accepted data source for population‐based cancer research and SEER covers >35% of the U.S. population.^10^ It provides detailed information on patient demographics, primary tumor site, tumor morphology, stage at diagnosis, and survival outcomes. The linkage to Medicare claims augments the data by supplying additional information on the health care utilization both before and after cancer diagnosis for cancer patients with Medicare insurance coverage including Parts A, B, and D. The Medicare Part D claims provide detailed information on pharmaceutical prescription use since 2007.

### 2.2 Study cohort

We identified patients aged 70 to 90 years newly diagnosed with AJCC Stage Group 6th Edition Stage I female breast cancer who had HR+/HER2− from the SEER‐Medicare database. Patients were diagnosed between 2010 and 2015 with claims till the end of 2016. We required that the patients had continuous enrollment in Medicare Parts A and B without health maintenance organization (HMO) coverage during the 12 months prior to diagnosis through 12 months after diagnosis to ensure complete records for identifying pre‐existing comorbidities, surgery, and radiation treatment during this timeframe. Further, we required that the patients had continuous enrollment in Medicare Part D during the 6 months after lumpectomy to ensure complete claims information to capture the utilization of endocrine therapy. We captured patients who received lumpectomy as their first treatment within 6 months of diagnosis and did not receive mastectomy based on Healthcare Common Procedure Coding System/Current Procedural Terminology (HCPCS/CPT) codes or International Classification of Diseases 9th Revision (ICD‐9) and 10th revision (ICD‐10) procedure codes. Breast cancer surgery billing codes for lumpectomy and mastectomy are provided in Appendix Table A1.

### 2.3 Utilization of radiation therapy and endocrine therapy

We identified patients without any use of radiation therapy based on HCPCS/CPT codes: 77402–77404, 77406–77409, 77411–77414, 77416, 77418, 77385–77386, 77761–77763, 77776–77778, 77785–77787, 77799, 0073T, 0182T. The utilization of ET was determined using the following generic prescription names: anastrozole, exemestane, letrozole, tamoxifen citrate, toremifene citrate and/or HCPCS/CPT code J9395.

### 2.4 Patient characteristics and outcome variables

The patient demographic, clinical, and socioeconomic characteristics included in this study were age at diagnosis, race, and ethnicity (White, Black, Hispanic, Asian, Other/Unknown), marital status (married, single, divorced/separated, widowed, other/unknown), urban/rural status (big metro, metro, urban, and less urban/rural), census tract median household income in quartiles, census tract percentage with college education or higher in quartiles, census tract percentage living below poverty in quartiles and Medicaid dual eligibility (yes, no). The big metro is central/fringe counties with population >1 million, metro is counties at metro area with 250,000 < population ≤1 million or metro area with population < 250,000, urban is urban population ≥ 20,000 adjacent or not adjacent to metro, less urban/rural is population between 19,999 to less than 2500. We calculated the Quan modification of the Charlson Comorbidity Index (CCI)^11^ based on all claims that occurred within 12 months before diagnosis via International Classification of Diseases 9th Revision (ICD‐9) and 10th revision (ICD‐10) diagnosis codes. We also included the year of breast cancer diagnosis in our analyses to capture time trend. One of the outcome variables we evaluated in this study was the use of ET after lumpectomy. If a patient had at least one claim of any of the aforementioned ET drugs within 6 months since lumpectomy, then she was defined as initiated endocrine therapy. In our study, we only evaluated the initiation of the endocrine therapy and not the adherence over time. The other outcome variable was patients' overall survival. The time window used for survival analysis was from 6 months after lumpectomy till the end of the study. The end of the study was defined as time of all‐cause death or till the end of the claims December 31, 2016. If a patient survived till the end of the study, the patient was considered censored on December 31, 2016 in the survival analyses. Cancer‐specific survival was not evaluated in this study as cancer‐specific survival data are not always accurate, especially in older patients.^12^

### 2.5 Statistical analyses

We provided sample descriptive statistics including frequencies and percentages stratified by ET initiation. Chi‐squared tests were used to test for subgroup differences in the use of ET by patient characteristics. A multivariable logistic regression model including all covariates with Chi‐squared *p*‐value <0.5 from the prior bivariate analyses was used to evaluate factors associated with ET initiation. We provided the odds ratios (ORs), the corresponding 95% confidence intervals (CIs) and p‐values for the parameters in the logistic model. We accessed the overall survival using Kaplan–Meier estimation and log‐rank test by the use of ET. Given that there could be confounding factors such as age,^13^ race and ethnicity, comorbidity,^14^ and other factors driving both the use of ET and the survival, we also conducted a Cox proportional hazards regression with inverse probability weighting. The selection of variables in estimating the propensity score was based on our review of the subject‐matter literature and expert opinion as suggested by Austin et al.^20^ Specifically, the weights were based on propensity scores calculated using the following variables: year of diagnosis, age at diagnosis, race and ethnicity,^15^, ^16^, ^17^ marital status at diagnosis, urban/rural code at diagnosis, Charlson comorbidity score, census tract percentage with some college education or higher, census tract percentage of residents living below poverty, and Medicaid dual‐eligible status.^18^, ^19^ The use of the inverse probability weighting approach would reduce the impact of the confounding factors on the overall survival. The hazard ratios (HRs), the corresponding 95% CIs and *p*‐values are provided.

All statistical tests were two‐sided, and *p*‐values were reported. We used significance level (alpha) at 0.05, 0.01, and 0.001 for statistical tests. We present the *p*‐values with the asterisk rating system to indicate the significance level for each statistical test. For example, * stands for statistically significant with *p*‐value<0.05, ** stands for statistically significant with *p*‐value<0.01, and *** stands for statistically highly significant with *p*‐value<0.001. The statistical analyses were conducted in SAS 9.4 (SAS Institute, Cary, NC). All patients were de‐identified in the SEER‐Medicare database, and the Institutional Review Board approved this retrospective observational study.

## 3 RESULTS

We identified a total of 2618 AJCC Stage I breast cancer patients aged 70 to 90 years who received lumpectomy and omitted radiation therapy. Of these patients, 1810 (69.1%) received ET during the 6 months after lumpectomy. Table 1 shows the characteristics of the study cohort stratified by whether ET was received. The percentage of patients who initiated endocrine therapy significantly increased from 62.6% in years 2010–2012 to 72.2% in 2013–2015. We also found significant differences in the use of ET by age, marital status, Charlson comorbidity score, and census tract percent of residents living below poverty.

**TABLE 1:** Association between initiation of endocrine therapy and patients' sociodemographic and clinical characteristics

Variable	Categories	No Endocrine therapy (*n* = 808) Row %	Endocrine therapy (*n* = 1810) Row %	All (*n* = 2618)	*p*‐value	Sig^a^
Derived AJCC Stage Group (6th edition)	Stage 1	30.9	69.1	2618	—	—
Year of diagnosis	2010–2012	37.4	62.6	846	<0.0001	***
	2013–2015	27.8	72.2	1772		
Age at diagnosis	70–80 year	23.8	76.2	1619	<0.0001	***
	81–90 year	42.2	57.8	999		
Race and ethnicity	White	31.1	68.9	2232	0.3105	
	Black	35.7	64.3	126		
	Hispanic	24.8	75.2	137		
	Asian	25.0	75.0	60		
	Other/Unknown	31.7	68.3	63		
Marital Status at diagnosis	Married	26.3	73.7	1038	0.0001	***
	Single, never married	28.7	71.3	247		
	Divorced/Separated	30.3	69.7	244		
	Widowed	35.6	64.4	949		
	Other/Unknown	37.1	62.9	140		
Urban/rural code at diagnosis^b^	Big metro	32.2	67.8	1413	0.3637	
	Metro	30.0	70.0	834		
	Urban	27.3	72.7	132		
	Less urban/Rural	28.0	72.0	239		
Charlson comorbidity score	0	29.8	70.2	1349	0.0482	*
	1	29.1	70.9	602		
	≥2	34.6	65.4	667		
Census Tract Median Household Income (quartiles)	Quartile 1 (≤$45540.0)	29.2	70.8	655	0.5245	
	Quartile 2 (>$45540.0 to ≤$61630.0)	32.1	67.9	654		
	Quartile 3 (>$61630.0 to ≤$85279.0)	29.9	70.1	655		
	Quartile 4 (>$85279.0)	32.3	67.7	654		
Census Tract Percent persons 25+ with some college education or higher (quartiles)	Quartile 1 (≤49.940)	29.4	70.6	654	0.3329	
	Quartile 2 (>49.940 to ≤64.885)	29.3	70.7	655		
	Quartile 3 (>64.885 to ≤77.400)	31.5	68.5	655		
	Quartile 4 (>77.400)	33.3	66.7	654		
Census Tract Percent of residents living below poverty (quartiles)	Quartile 1 (≤4.860)	29.3	70.7	656	0.0143	*
	Quartile 2 (>4.860 to ≤8.940)	35.6	64.4	654		
	Quartile 3 (>8.940 to ≤15.630)	30.7	69.3	654		
	Quartile 4 (>15.630)	27.8	72.2	654		
Medicaid dual‐eligible	No	30.4	69.6	2152	0.2603	
	Yes	33.0	67.0	466		
Tumor size	≥0 to <10 mm	31.3	68.7	1181	0.6397	
	≥10 mm	30.5	69.5	1437		

*Note*: Chi‐square Test was used to obtain all *p*‐values.

Abbreviation: AJCC, American Joint Committee on Cancer.

^a^ Sig is the significance level, * stands for statistically significant at significance level of alpha = 0.05, and *** stands for statistically highly significant with significance level of alpha = 0.001.

^b^ Urban/rural code at diagnosis: big metro is central/fringe counties with population >1 million, metro is counties at metro area with 250,000 < population ≤1 million or metro area with population < 250,000, urban is urban population ≥ 20,000 adjacent or not adjacent to metro, less urban/rural is population between 19,999 and less than 2500.

Table 2 provides the results from the multivariable logistic regression model. The estimates indicate a significant increase in the uptake of ET in 2013–2015 compared to 2010–2012 with an OR of 1.39 (95%CI = [1.16–1.66], *p*‐value<0.001). Older age (81–90 vs. 70–80 years of age) was associated with significantly lower odds (OR = 0.45, 95%CI = [0.38,0.54], *p*‐value<0.001) of receiving ET.

**TABLE 2:** Multivariable logistic regression to evaluate factors associated with initiation of endocrine therapy among breast cancer patients

Variable	Categories	Odds ratio estimate	Odds ratio 95% CI	Pr > Chi‐Square	Sig^a^
Year of diagnosis	2010–2012	Reference	—	—	
	2013–2015	1.39	[1.16,1.66]	<0.001	***
Age at diagnosis	70‐80 year	Reference	—	—	
	81–90 year	0.45	[0.38,0.54]	<0.001	***
Race and Ethnicity	White	Reference	—	—	
	Black	0.77	[0.51,1.16]	0.208	
	Hispanic	1.27	[0.83,1.93]	0.265	
	Asian	1.62	[0.87,3.02]	0.13	
	Other/Unknown	0.89	[0.51,1.55]	0.674	
Marital Status at diagnosis	Married	Reference	—	—	
	Single, never married	0.94	[0.68, 1.30]	0.705	
	Divorced/Separated	0.88	[0.64, 1.21]	0.422	
	Widowed	0.85	[0.69, 1.04]	0.11	
	Other/Unknown	0.66	[0.45, 0.97]	0.033	*
Urban/rural code at diagnosis^b^	Big metro	Reference	—	—	
	Metro	1.1	[0.90, 1.33]	0.345	
	Urban	1.17	[0.77, 1.79]	0.452	
	Less urban/Rural	1.15	[0.83, 1.61]	0.404	
Charlson comorbidity score	0	Reference	—	—	
	1	1.03	[0.83, 1.28]	0.788	
	≥2	0.9	[0.73, 1.11]	0.344	
Census Tract Percent persons 25+ with some college education or higher (quartiles)	Quartile 1 (≤49.940)	Reference	—	—	
	Quartile 2 (>49.940 to ≤64.885)	1.07	[0.82, 1.39]	0.632	
	Quartile 3 (>64.885 to ≤77.400)	0.94	[0.71, 1.24]	0.662	
	Quartile 4 (>77.400)	0.84	[0.63, 1.13]	0.249	
Census Tract Percent of residents living below poverty (quartiles)	Quartile 1 (≤4.860)	Reference	—	—	
	Quartile 2 (>4.860 to ≤8.940)	0.69	[0.54,0.87]	0.002	**
	Quartile 3 (>8.940 to ≤15.630)	0.85	[0.66,1.10]	0.22	
	Quartile 4 (>15.630)	1.03	[0.77,1.39]	0.843	
Medicaid dual‐eligible	No	Reference	—	—	
	Yes	0.85	[0.66,1.09]	0.191	

^a^ Sig is the significance level, * stands for statistically significant at significance level of alpha = 0.05, ** stands for statistically significant at significance level of alpha = 0.01, and *** stands for statistically highly significant with significance level of alpha = 0.001.

^b^ Urban/rural code at diagnosis: big metro is central/fringe counties with population >1 million, metro is counties at metro area with 250,000 < population ≤1 million or metro area with population < 250,000, urban is urban population ≥ 20,000 adjacent or not adjacent to metro, less urban/rural is population between 19,999 and less than 2500.

**FIGURE 1:**
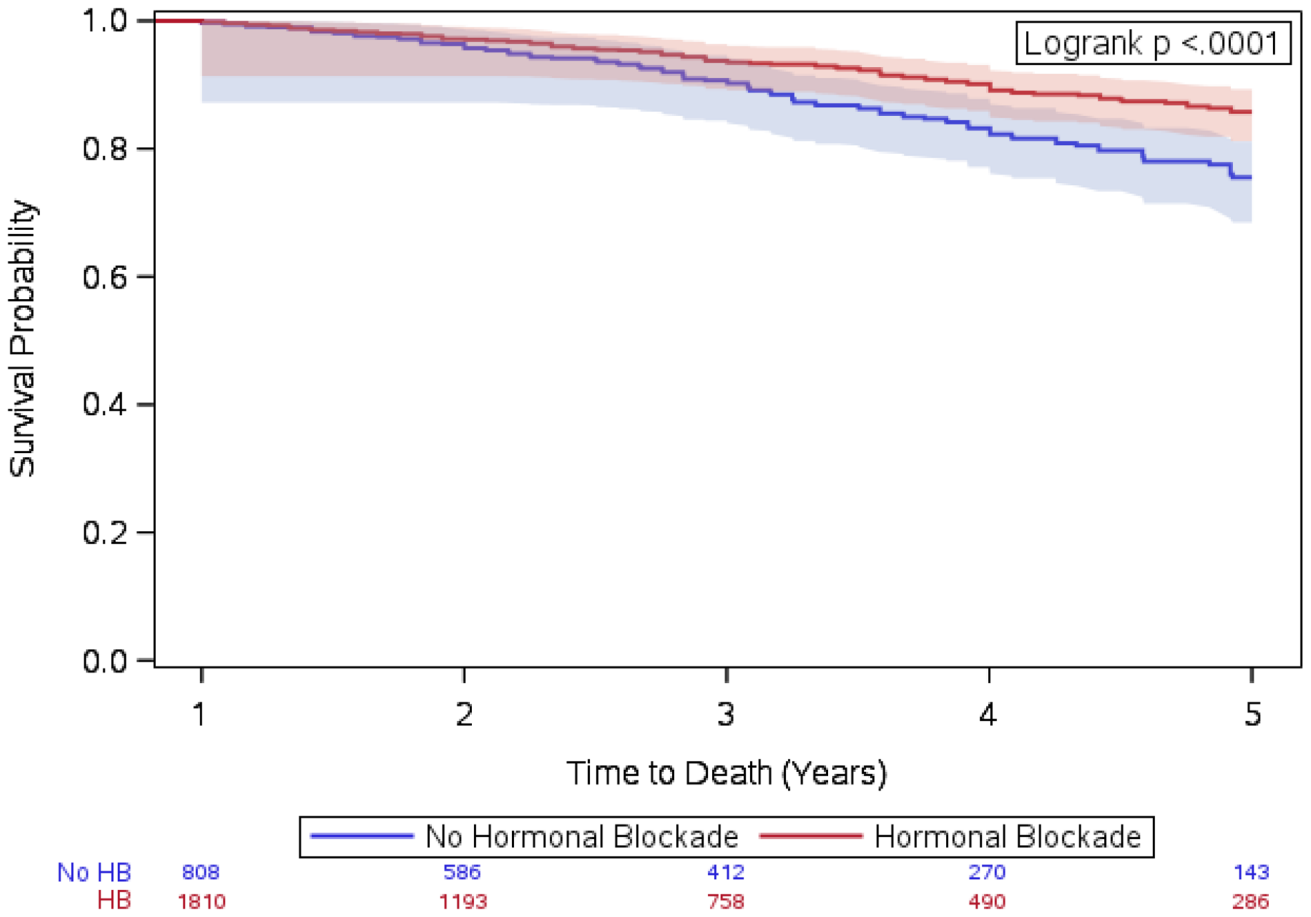
Kaplan–Meier curves for overall survival by endocrine therapy use.

Kaplan–Meier curves and log‐rank test results are shown in Figure 1. The overall survival of patients receiving ET was significantly better than patients not receiving it (*p*‐value<0.0001) based on the log‐rank test. We also conducted a Cox proportional hazard model with inverse probability weighting based on the propensity scores obtained using the multivariable logistic regression model as aforementioned. After the weighting, the absolute standardized mean difference was less than 0.005 which indicates that the confounding variables were adjusted well in the ET and non‐ET groups. Table 3 includes the detailed results from the weighted Cox proportional hazard regression. We found that patients who received endocrine therapy had significantly better survival than patients who did not receive it (HR = 0.76, 95%CI = [0.58,0.99], *p*‐value = 0.04). We also found that more recent years (2013–2015 compared to 2010–2012) were associated with better survival (HR = 0.70, 95%CI = [0.49,0.98], *p*‐value = 0.04). As expected, older age (81 = 90 vs. 70–80: HR = 2.30, 95%CI = [1.71,3.09], *p*‐value<0.001) was associated with worse survival. Similarly, higher comorbidities were associated with worse overall survival (1 vs. 0: HR = 1.98, 95%CI = [1.32,2.97], *p*‐value = 0.001, and 2+ vs. 0: HR = 3.11, 95%CI = [2.26, 4.29], *p*‐value<0.001). We also found marital status and urban/rural status to be associated with overall survival.

**TABLE 3:** Factors associated with patients' overall survival based on inverse probability weighted multivariable Cox proportional Hazard model

Parameter	Comparison	Hazard ratio	Hazard ratio	Pr > Chi‐Square	Sig^a^
			95% CI		
Endocrine therapy	No	Reference	—	—	
	Yes	0.76	[0.58,0.99]	0.04	*
Year of diagnosis	2010–2012	Reference	—	—	
	2013–2015	0.7	[0.49,0.98]	0.04	*
Age at diagnosis	70–80 year	Reference	—	—	
	81–90 year	2.3	[1.71,3.09]	<0.001	***
Race and Ethnicity	White	Reference	—	—	
	Black	1.02	[0.54,1.93]	0.955	
	Hispanic	0.48	[0.23,1.01]	0.054	
	Asian	0.97	[0.37,2.54]	0.952	
	Other/Unknown	0.72	[0.27,1.95]	0.52	
Marital Status at diagnosis	Married	Reference	—	—	
	Single, never married	1.96	[1.13, 3.40]	0.017	*
	Divorced/Separated	1.35	[0.77, 2.35]	0.297	
	Widowed	1.69	[1.18, 2.42]	0.004	**
	Other/Unknown	2.11	[1.16, 3.83]	0.015	*
Urban/rural code at diagnosis^b^	Big Metro	Reference	—	—	
	Metro	1.42	[1.04, 1.95]	0.029	*
	Urban	1.57	[0.84, 2.91]	0.154	
	Less urban/Rural	1.73	[1.09, 2.76]	0.021	*
Charlson comorbidity score	0	Reference	—	—	
	1	1.98	[1.32, 2.97]	0.001	**
	≥ 2	3.11	[2.26, 4.29]	<0.001	***
Census Tract Percent of residents living below poverty (quartiles)	Quartile 1 (≤4.860)	Reference	—	—	
	Quartile 2 (>4.860 to ≤8.940)	1.3	[0.91,1.87]	0.152	
	Quartile 3 (>8.940 to ≤15.630)	1.05	[0.67,1.65]	0.832	
	Quartile 4 (>15.630)	1.1	[0.70,1.75]	0.677	
Census Tract Percent persons 25+ with some college education or higher (quartiles)	Quartile 1 (≤49.940)	Reference	—	—	
	Quartile 2 (>49.940 to ≤64.885)	1.05	[0.72, 1.54]	0.806	
	Quartile 3 (>64.885 to ≤77.400)	1.2	[0.77, 1.86]	0.415	
	Quartile 4 (>77.400)	1.02	[0.63, 1.67]	0.933	
Medicaid dual‐eligible	No	Reference	—	—	
	Yes	1.3	[0.91,1.87]	0.152	

^a^ Sig is the significance level, * stands for statistically significant at significance level of alpha = 0.05, ** stands for statistically significant at significance level of alpha = 0.01, and *** stands for statistically highly significant with significance level of alpha = 0.001.

^b^ Urban/rural code at diagnosis: big metro is central/fringe counties with population >1 million, metro is counties at metro area with 250,000 < population ≤1 million or metro area with population < 250,000, urban is urban population ≥ 20,000 adjacent or not adjacent to metro, less urban/rural is population between 19,999 and less than 2500.

## 4 DISCUSSION

This study examined the initiation of ET in older women with early‐stage HR+/HER2− breast cancer who received breast‐conserving surgery but omitted radiation therapy. Further, it also examined the impact of ET use on survival outcomes when radiation therapy was not delivered. Coinciding with the decreased utilization rate of radiation therapy over time revealed in the literature,^5^, ^6^ our findings showed the percentage of patients with ET use significantly increased from 62.6% in patients diagnosed in years 2010–2012 to 72.2% in 2013–2015. Recent trends showed higher uptake of adjuvant ET suggests that clinicians are becoming more aware of the importance of ET especially among patients who omitted radiation. The overall survival analyses suggested that the use of ET was associated with significantly better survival, providing valuable evidence to support the recommended guidelines that older patients should receive ET after forgoing radiation therapy.^9^, ^21^, ^22^

In our study, we observed increased use of ET over time, which in part may be due to the improvement in physicians' compliance to clinical practice guidelines. In addition, Affordable Care Act (ACA) enacted in 2010 may have played an important role in the improvement in ET uptake in breast cancer patients. The implementation of ACA has helped over 20 million Americans gain health care coverage and allowed many cancer patients to have access to standard cancer care.^23^ However, there is still large room for improvement for all eligible patients to receive ET, especially for women >80 years. Our finding of older age is associated with less use of ET is consistent with the literature showing lower odds of adherence to endocrine therapy among older women.^22^, ^24^ This is possibly due to the older patients more likely to have shortened life expectancy, competing comorbidities, and side effects such as headache, depression, and musculoskeletal symptoms.^25^, ^26^, ^27^ Such side effects present significant challenges to physicians when managing treatment for older patients. Studies have found that patients ≥70 years old with comorbidity are a common reason for ET to be declined and/or not recommended by physicians.^28^, ^29^ In order to improve ET initiation, researchers have evaluated various strategies such as using patient navigator, providing bilingual and cultural tailored approach, using smartphone app‐based approach, and designing patient decision aids to improve ET initiation and decrease discontinuation.^30^, ^31^, ^32^ Some of these strategies will be useful to help older patients who omit radiation therapy to initiate ET.

Our study is subject to several limitations. First, although Medicare Part D claims data provide detailed information on filled pharmaceutical prescriptions, it cannot be ascertained whether the prescriptions were actually taken in the prescribed fashion by the patients. Second, the SEER‐Medicare data used in this study lack information on the reason why the patients did not receive ET. Information on potential reasons for non‐initiation such as surgical complications and side effects are not available in the database. Future studies could survey providers and patients as to the reasons why ET was not initiated or taken. Third, the patients in this study were all Medicare enrollees with fee‐for‐service program of Part A (inpatient/hospital coverage), Part B (outpatients/medical coverage), and Part D (prescription drug coverage) insurance, making it difficult to generalize the findings to other patient groups such as patients with HMO coverage or private prescription drug insurance coverage. Fourth, this study examines the ET use within the 6 months after lumpectomy, which primarily focuses on the immediate initiation and its impacts on survival. Other than uptake of ET, another important research direction is the adherence over time to the recommended endocrine therapy.^22^, ^33^ Since our claims data were ended in 2016, the follow‐up time for our study cohort was not sufficiently long to evaluate the long‐term association between endocrine therapy adherence and patients' survival benefit. An updated analysis with longer follow‐up would provide more information on the association between ET adherence and survival.

Nevertheless, this is the first nationwide large observational study in the literature to examine both the initiation of ET and the corresponding survival outcomes when radiation therapy was omitted after lumpectomy in older women with early‐stage HR+/HER2‐ breast cancer. It revealed that a substantial proportion of the patients did not receive the recommended ET, and that the double omission of radiation and ET was associated with inferior survival outcomes.

## AUTHOR CONTRIBUTIONS

**Chan Shen:** Conceptualization (lead); data curation (lead); formal analysis (equal); funding acquisition (equal); investigation (lead); methodology (lead); project administration (lead); resources (lead); supervision (lead); validation (equal); visualization (equal); writing – original draft (lead); writing – review and editing (lead). **Ning Li:** Conceptualization (supporting); data curation (supporting); formal analysis (supporting); funding acquisition (supporting); investigation (supporting); methodology (equal); project administration (equal); resources (equal); software (equal); supervision (supporting); validation (equal); visualization (equal); writing – original draft (supporting); writing – review and editing (supporting). **Shouhao Zhou:** Conceptualization (equal); data curation (equal); formal analysis (equal); funding acquisition (equal); investigation (equal); methodology (equal); project administration (equal); resources (equal); software (equal); supervision (equal); validation (equal); visualization (equal); writing – original draft (equal); writing – review and editing (equal). **Kelly Stahl:** Conceptualization (equal); data curation (equal); formal analysis (equal); funding acquisition (equal); investigation (equal); methodology (equal); project administration (equal); resources (equal); software (equal); supervision (equal); validation (equal); visualization (equal); writing – original draft (equal); writing – review and editing (equal). **Daleela Dodge:** Conceptualization (equal); data curation (equal); formal analysis (equal); funding acquisition (equal); investigation (equal); methodology (equal); project administration (equal); resources (equal); software (equal); supervision (equal); validation (equal); visualization (equal); writing – original draft (equal); writing – review and editing (equal). **Hui Zhao:** Conceptualization (lead); data curation (equal); formal analysis (equal); funding acquisition (equal); investigation (lead); methodology (lead); project administration (equal); resources (equal); software (equal); supervision (equal); validation (equal); visualization (equal); writing – original draft (equal); writing – review and editing (equal).

## ETHICS STATEMENT

This study received expedited approval from the Institutional review board because the data are de‐identified.

## Data Availability

The datasets generated for this study are based on SEER‐Medicare data with cancer cases diagnosed between 2007‐2015 and claims till December 2016. The data can be purchased through the National Cancer Institute: https://healthcaredelivery.cancer.gov/seermedicare/obtain/cost.html.

## Appendix

**TABLE A1:** Billing codes used to define breast cancer surgery

Surgery	ICD‐9 Procedure Codes	ICD‐10 Procedure Codes	CPT/HCPCS Codes
Mastectomy			
	85.33, 85.34, 85.40, 85.41,85.42, 85.43, 85.45, 85.47	0HTT0ZZ, 0HTU0ZZ, 07T50ZZ, 07T60ZZ, 07T70ZZ, 07T80ZZ, 07T90ZZ, 0KTH0ZZ, 0KTJ0ZZ	19180, 19182, 19200, 19220, 19240, 19303, 19304, 19305, 19306, 19307
Lumpectomy			
	85.20, 85.21, 85.22, 85.23, 85.25	0H5T0ZZ, 0H5T3ZZ, 0H5T7ZZ, 0H5T8ZZ, 0H5TXZZ, 0H5U0ZZ, 0H5U3ZZ, 0H5U7ZZ, 0H5U8ZZ, 0H5UXZZ, 0H5V0ZZ, 0H5V3ZZ, 0H5V7ZZ, 0H5V8ZZ, 0H5VXZZ, 0HBT0ZZ, 0HBT3ZZ, 0HBT7ZZ, 0HBT8ZZ, 0HBTXZZ, 0HBU0ZZ, 0HBU3ZZ, 0HBU7ZZ, 0HBU8ZZ, 0HBUXZZ, 0HBV0ZZ, 0HBV3ZZ, 0HBV7ZZ, 0HBV8ZZ, 0HBVXZZ, 0H5W0ZZ, 0H5W3ZZ, 0H5W7ZZ, 0H5W8ZZ, 0H5WXZZ, 0H5X0ZZ, 0HBW0ZZ, 0HBW3ZZ, 0HBW7ZZ, 0HBW8ZZ, 0HBWXZZ, 0HBX0ZZ, 0HBX3ZZ, 0HBX7ZZ, 0HBX8ZZ, 0HBXXZZ, 0HTWXZZ, 0HTXXZZ	19110, 19120, 19125, 19126, 19160, 19162, 19301, 19302
